# Wide-area antral pulmonary vein and posterior wall isolation by way of segmental nonocclusive applications using a novel radiofrequency ablation balloon

**DOI:** 10.1016/j.hrcr.2021.02.005

**Published:** 2021-07-27

**Authors:** Arash Aryana, Shelley L. Allen, Luigi Di Biase, Mark R. Bowers, Padraig Gearoid O’Neill, Andrea Natale

**Affiliations:** ∗ Mercy General Hospital and Dignity Health Heart and Vascular Institute, Sacramento, California; † Albert Einstein College of Medicine and Montefiore Medical Center, Bronx, New York; ‡ Texas Cardiac Arrhythmia Institute, St. David’s Medical Center, Austin, Texas

**Keywords:** Atrial fibrillation, Catheter ablation, HelioStar, LassoStar, Nonocclusive, Posterior wall, Pulmonary vein isolation, Radiofrequency balloon, RF

## Introduction

Pulmonary vein (PV) isolation (PVI) remains the cornerstone of catheter ablation of atrial fibrillation (AF)^1^ and prior studies^2^^,^^3^ have shown that wide-area, antral PV ablation is superior to ostial PVI. Yet, an inherent limitation of balloon-based AF ablation strategies is that they frequently yield an ostial-level PVI.^4^ Thus, for optimal results and to achieve wide-area, antral PVI, segmental non-PV occlusive applications are commonly required. Such an approach not only can yield an antral-level PVI, but it allows the operator to perform extra-PV ablation, such as posterior wall isolation (PWI). In this manuscript, the authors describe the first reported case of PVI with concomitant PWI performed by means of segmental non-PV occlusive applications using the novel radiofrequency (RF) ablation balloon (HelioStar; Biosense Webster, Irvine, CA), under the direct visualization of 3-D mapping and guided by electrode impedance.

**Key Teaching Points**

• An inherent limitation of balloon-based atrial fibrillation ablation strategies is that they frequently yield pulmonary vein (PV) isolation (PVI) at an ostial level.
• Thus, to achieve wide-area, antral PVI, segmental non-PV occlusive applications are commonly required.
• Such an approach not only can yield an antral-level PVI, but it allows the operator to perform extra-PV ablation, such as posterior wall isolation (PWI).
• As illustrated in this manuscript, wide-area, antral PVI with concomitant PWI can be safely and effectively performed with the novel HelioStar (Biosense Webster, Irvine, CA) radiofrequency ablation balloon using a segmental non-PV occlusive approach, guided by 3-D mapping and impedance.

## Case report

**Figure 1:**
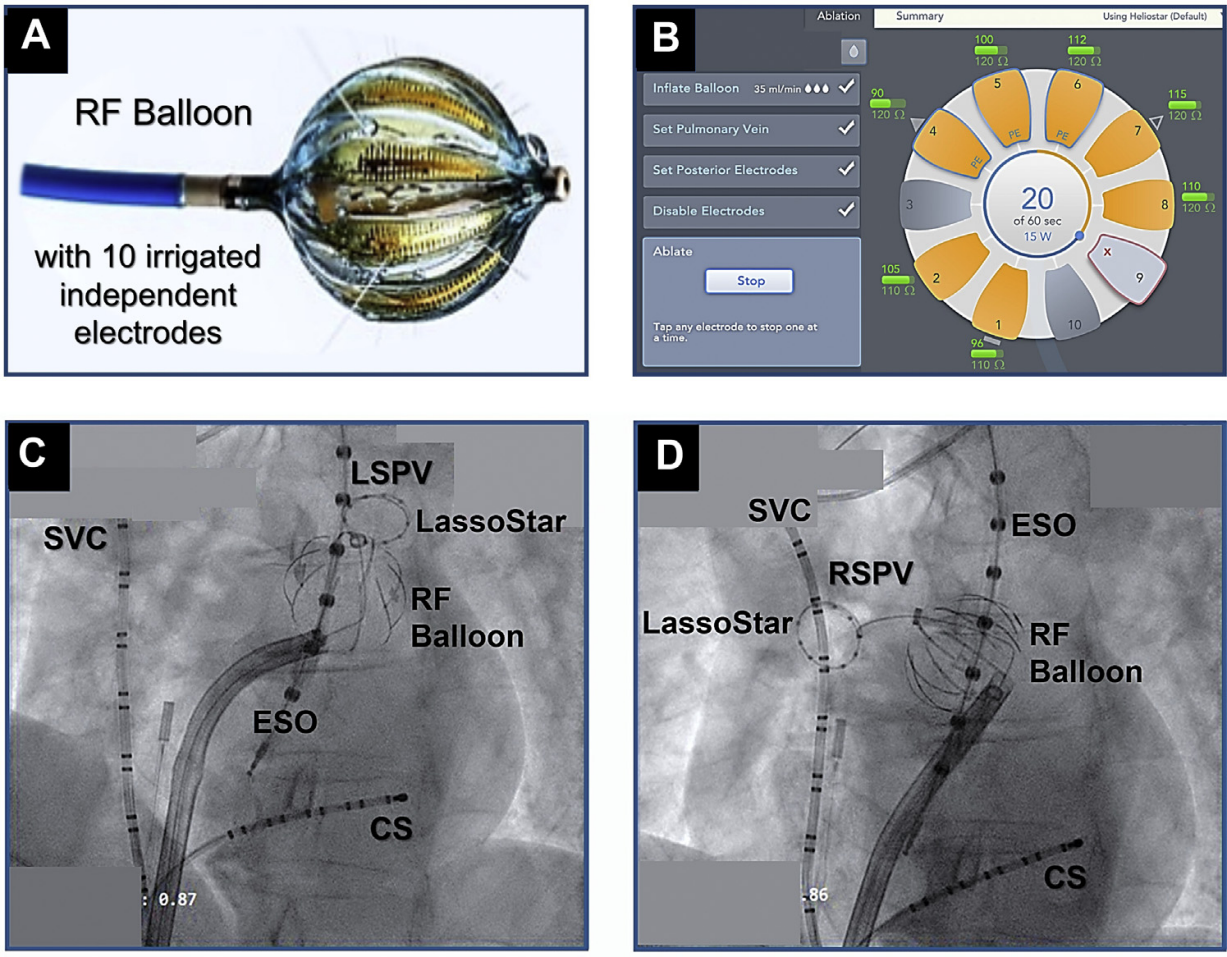
The radiofrequency (RF) balloon and segmental non–pulmonary vein (PV) occlusive applications. **A:** The HelioStar (Biosense Webster, Irvine, CA) RF ablation balloon is 28 mm in diameter with 10 irrigated electrodes and 4 irrigation ports per electrode. Each electrode is 14.5 mm long with a maximum width of 4.1 mm and a variable spacing of 4.1–5.6 mm. A multielectrode generator allows the operator to independently control the duration and energy delivered from each of the 10 electrodes **B:** To achieve wide-area, antral PV isolation, PV occlusion may be avoided and the PVs are instead targeted using a segmental nonocclusive approach. **C:** A cine demonstrating noncoaxial placement of the RF balloon along the anterior aspect of the left superior PV (LSPV) for ablation of the superior segment of the left atrial (LA) ridge. The balloon is placed over a 3F, fixed-loop, 20-mm, 10-pole circular mapping catheter (LassoStar; Biosense Webster). Additionally, a decapolar catheter is seen inside the coronary sinus (CS), a duodecapolar catheter in the superior vena cava (SVC), and an esophageal temperature probe within the esophagus (ESO). **D:** A cine illustrating placement of the RF balloon on the posterior wall, outside of the right superior PV (RSPV) ostium, to perform antral ablation of the superior segment of the LA posterior wall.

A 76-year-old woman with past medical history significant for recurrent symptomatic paroxysmal AF refractory to antiarrhythmic therapy with sotalol and flecainide and multiple cardioversions with early recurrence following the last attempt, in the setting of sleep apnea and pulmonary hypertension, underwent catheter ablation using the novel RF ablation balloon while receiving uninterrupted oral anticoagulation therapy. The procedure was performed under general anesthesia. Bilateral femoral venous access was obtained under ultrasound guidance. A decapolar catheter was inserted inside the coronary sinus for left atrial (LA) recording and pacing and a duodecapolar catheter was inserted in the high right atrium/superior vena cava for right atrial recording and pacing and phrenic nerve (PN) stimulation. After intravenous systemic anticoagulation, a transseptal puncture was performed using the conventional method. Next, the LA and the 4 PVs were mapped by fast anatomical mapping (Carto; Biosense Webster) using a 3F, diagnostic, inner-lumen circular mapping catheter (LassoStar; Biosense Webster), introduced through the RF balloon, which was in turn inserted via a unidirectional 13.5F introducer (GuideStar; Biosense Webster). The ablation catheter is a compliant, multielectrode, 28-mm RF balloon with 10 irrigated electrodes (4 irrigation ports/electrode) that allows delivery of unipolar RF energy from selected electrodes circumferentially or in a segmental fashion (Figure 1A). The electrodes are long, spanning 14.5 mm longitudinally along the surface of the balloon, rendering it suitable for non-PV occlusive applications when placed in parallel to the tissue and targeting non-PV structures such as the posterior wall. A multielectrode generator allows the operator to independently control the duration and energy (power) delivered from each of the 10 electrodes (Figure 1B). Meanwhile, the ablation catheter is fully integrated with the 3-D mapping system (Carto; Biosense Webster), which allows direct visualization of the RF balloon and the inner-lumen circular mapping catheter within the 3-D map. The latter consists of a 3F, 20-mm, fixed-loop, circular catheter with 10 evenly spaced 1-mm electrodes. In addition to allowing for validation of real-time to PVI, it can be used to create a 3-D map by fast anatomical mapping.

**Figure 2:**
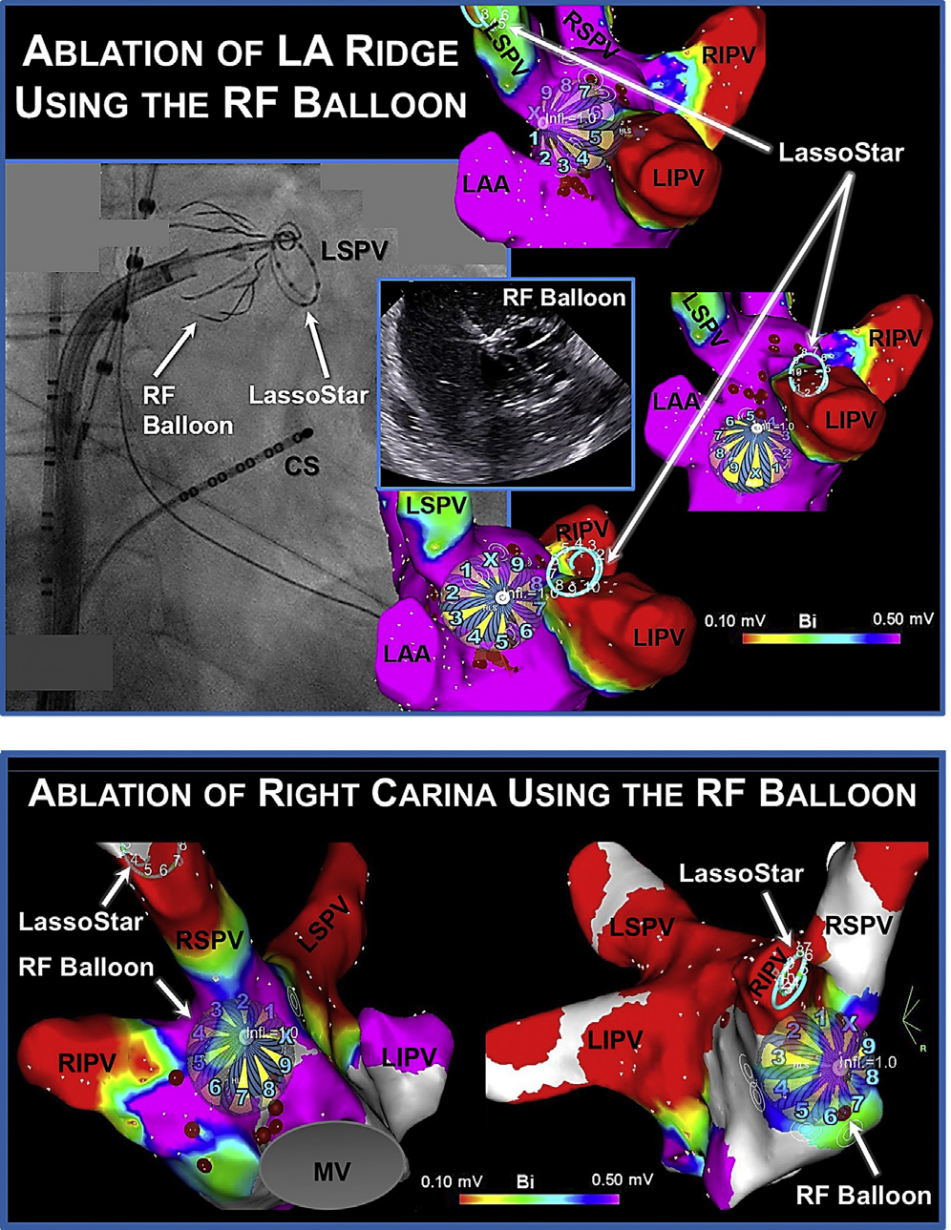
Ablation of the left atrial (LA) ridge and the carina. **Top panel:** Cine and intracardiac echocardiographic images and 3-D bipolar voltage maps of the LA depicting the positions of the radiofrequency (RF) balloon along the ridge between the left pulmonary veins (PVs) and the appendage. The RF balloon is placed over a 3F, 10-pole inner-lumen circular mapping catheter (LassoStar; Biosense Webster, Irvine, CA), used as a rail to navigate and control the placement of the balloon. **Lower panel:** 3-D LA voltage maps illustrating the positions of the RF balloon along the anterior carina adjacent to the right PVs, placed over the inner-lumen mapping catheter. RF balloon placement is guided by 3-D mapping and repositioning of the inner-lumen catheter inside the right superior and the right inferior PVs. CS = coronary sinus; LAA = LA appendage; LIPV = left inferior PV; LSPV = left superior PV; MV = mitral valve; RIPV = right inferior PV; RSPV = right superior PV.

**Figure 3:**
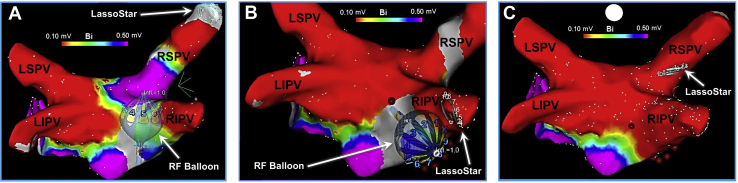
Radiofrequency (RF) balloon–guided pulmonary vein (PV) isolation and posterior wall isolation (PWI). Shown, are progressive 3-D bipolar voltage maps of the left atrium (LA) depicting the positions of the RF balloon during segmental non-PV occlusive applications along the posterior wall, targeting the antral portions of the right superior (**A**) and inferior (**B**) PVs. Once again, positioning of the RF balloon is guided by 3-D mapping and the inner-lumen circular mapping catheter. As seen, this approach can yield wide-area, antral PV isolation along with PWI within the PV component (ie, the region of the LA posterior wall that lies between the PVs). **C:** A 3-D voltage map of the LA created postablation using a high-density, multielectrode, diagnostic mapping catheter (PentaRay; Biosense Webster, Irvine, CA), validating PV isolation and PWI (voltage cutoff: 0.1 mV). LIPV = left inferior PV; LSPV = left superior PV; RIPV = right inferior PV; RSPV = right superior PV.

In the current patient, all 4 PVs were ablated antrally using the HelioStar RF balloon through a series of non-PV occlusive applications, guided by 3-D mapping. Ablation was performed using 15 watts for 60 seconds from electrodes projecting on the anterior wall and for 30 seconds from those on the posterior wall of the LA. An activated clotting time >350 seconds was maintained during ablation and luminal esophageal temperature monitoring was also performed. Esophageal temperature rises >1°C above baseline were avoided. Additionally, when targeting the right PVs, continuous high-output stimulation (25 mA; 1000 ms) was performed using the duodecapolar catheter inside the superior vena cava. At no time was there loss of right PN pacing capture. Since during ablation PV occlusion was not attempted, no contrast medium injection was required. Instead, the PVs were targeted and segmentally isolated using a nonocclusive strategy (Figure 1C and 1D). The inner-lumen circular mapping catheter was used as a rail to navigate and control the placement of the RF balloon. Similarly, the LA ridge on the left (the area between the PVs and the appendage) and the carina on the right were targeted using antral, segmental applications (Figure 2). The approach was primarily guided and greatly facilitated by 3-D mapping, which allows direct visualization of the balloon and the inner-lumen circular mapping catheter in the LA map. During segmental ablation, RF applications were delivered from the electrodes that were in optimal tissue contact. Optimal electrode–tissue contact during nonocclusive applications to the PV antra / posterior wall was assessed by examining the electrode impedance (Supplemental Video). Both the baseline electrode impedance and temperature can be used to differentiate optimal (>95 Ω, <30°C) vs suboptimal tissue contact (<80 Ω, >32°C) and to guide individual electrode selection during RF delivery. Altogether, 13 RF applications and 10 minutes and 9 seconds of ablation time was required to achieve bilateral, wide-area, antral PVI as well as PWI within the region of the PV component^5^—ie, the LA posterior wall region lying between the PVs (Figure 3). As such, the posterior wall itself was directly ablated and “homogenized” using the balloon as opposed to performing linear ablations. This was further validated by 3-D mapping using a high-density, multielectrode, diagnostic mapping catheter (PentaRay; Biosense Webster), postablation (Figure 3C). Furthermore, PVI+PWI was also confirmed by performing high-output pacing (>10 mA) extensively, from multiple sites within the area of isolation. At the completion of the procedure, the patient was extubated and discharged to home on the following day. She tolerated the procedure well without any acute or long-term adverse events, including no PN-, vagal-, or esophagus-related complications. She has maintained sinus rhythm during 14 months of follow-up, off antiarrhythmic therapy. The follow-up consisted of weekly, 60-second, transtelephonic monitoring during the first 6 months, followed by monthly transmissions from 6–12 months and a 24-hour Holter monitor at 12 months, in addition to routine 12-lead electrocardiograms obtained during 1-, 3-, 6-, and 12-month follow-up visits.

## Discussion

Several studies have shown a marked benefit associated with an antral- versus an ostial-level PVI.^2^^,^^3^ In a meta-analysis of 12 studies including 1183 AF patients treated with ostial vs antral PVI, AF recurrence was significantly lower in those treated with a wide, antral approach, with an odds ratio of 0.33 (95% confidence interval: 0.24–0.46; *P* < .00001).^3^ Although prior studies have failed to show a significant advantage with additional substrate-based modification, recent studies^6^, ^7^, ^8^ have found a notable benefit associated with PWI when performed in conjunction with PVI within the region of the PV component,^5^ particularly in patients with persistent AF. Although wide, antral PVI and PWI can be successfully performed using point-by-point RF, retrospective^9^^,^^10^ and prospective randomized trials^11^ of PVI+PWI using balloon-based strategies have consistently found this approach to be superior to PVI alone. But this approach requires delivery of segmental non-PV occlusive applications. Aside from eliminating the need for contrast medium use/injection, a non-PV occlusive approach likely also minimizes the risk of PN injury. To elaborate, the reason why PN palsy occurs more commonly with balloon-based AF ablation strategies—be it using the cryoballoon,^12^ the laser balloon,^12^ or the HelioStar balloon^13^ as compared to point-by-point RF^14^—has to do with a more distal placement of balloon catheters inside the right PV ostia during ablation, particularly the right superior PV, which often not only exhibits a larger ostium, but lies closest to the right PN. This also sheds light on the relatively low incidence of PN palsy associated with the laser balloon,^12^ which can expand to a larger diameter (up to 38 mm), as compared to the much smaller (23-, 25-, or 28-mm) cryoablation and RF ablation balloons.^12^^,^^13^^,^^15^

## Conclusion

As illustrated in this initial report, wide-area, antral PVI with concomitant PWI can be safely and effectively performed with the novel HelioStar RF balloon using a segmental non-PV occlusive approach, under direct visualization within the 3-D map and guided by impedance. Future studies are necessary to validate the efficacy of this strategy as well as its perceived safety as compared to conventional PV occlusion–guided ablation in patients with AF.

## Appendix Supplementary data

Supplemental Video Biophysical assessment of radiofrequency balloon electrode–tissue contact. Top panel, A 3-D map of the LA depicting deliberate movements of the RF balloon along the posterior antrum of the left PVs over the inner-lumen circular mapping catheter (LassoStar), which is placed inside the left superior PV as a rail/anchor. Lower panel, A live display of the balloon electrode impedance measurements varying with changes in electrode–tissue contact. Baseline electrode impedance as well as temperature may be used to differentiate optimal versus suboptimal/poor tissue contact and to guide electrode selection for RF delivery during segmental applications. LIPV=left inferior PV; LSPV=left superior PV; RIPV=right inferior PV; RSPV=right superior PV.
