# Supplementary material for: Wide-area antral pulmonary vein and posterior wall isolation by way of segmental nonocclusive applications using a novel radiofrequency ablation balloon
Source: HeartRhythm Case Rep. 2021 Jul 27;7(8):518–21. doi: 10.1016/j.hrcr.2021.02.005 (PMC8377238; doi:10.1016/j.hrcr.2021.02.005)
Supplement: Supplemental Video — Biophysical assessment of radiofrequency balloon electrode–tissue contact. Top panel, A 3-D map of the LA depicting deliberate movements of the RF balloon along the posterior antrum of the left PVs over the inner-lumen circular mapping catheter (LassoStar), which is placed inside the left superior PV as a rail/anchor. Lower panel, A live display of the balloon electrode impedance measurements varying with changes in electrode–tissue contact. Baseline electrode impedance as well as temperature may be used to differentiate optimal versus suboptimal/poor tissue contact and to guide electrode selection for RF delivery during segmental applications. LIPV=left inferior PV; LSPV=left superior PV; RIPV=right inferior PV; RSPV=right superior PV. [file mmc1.pptx]

## Slide 1
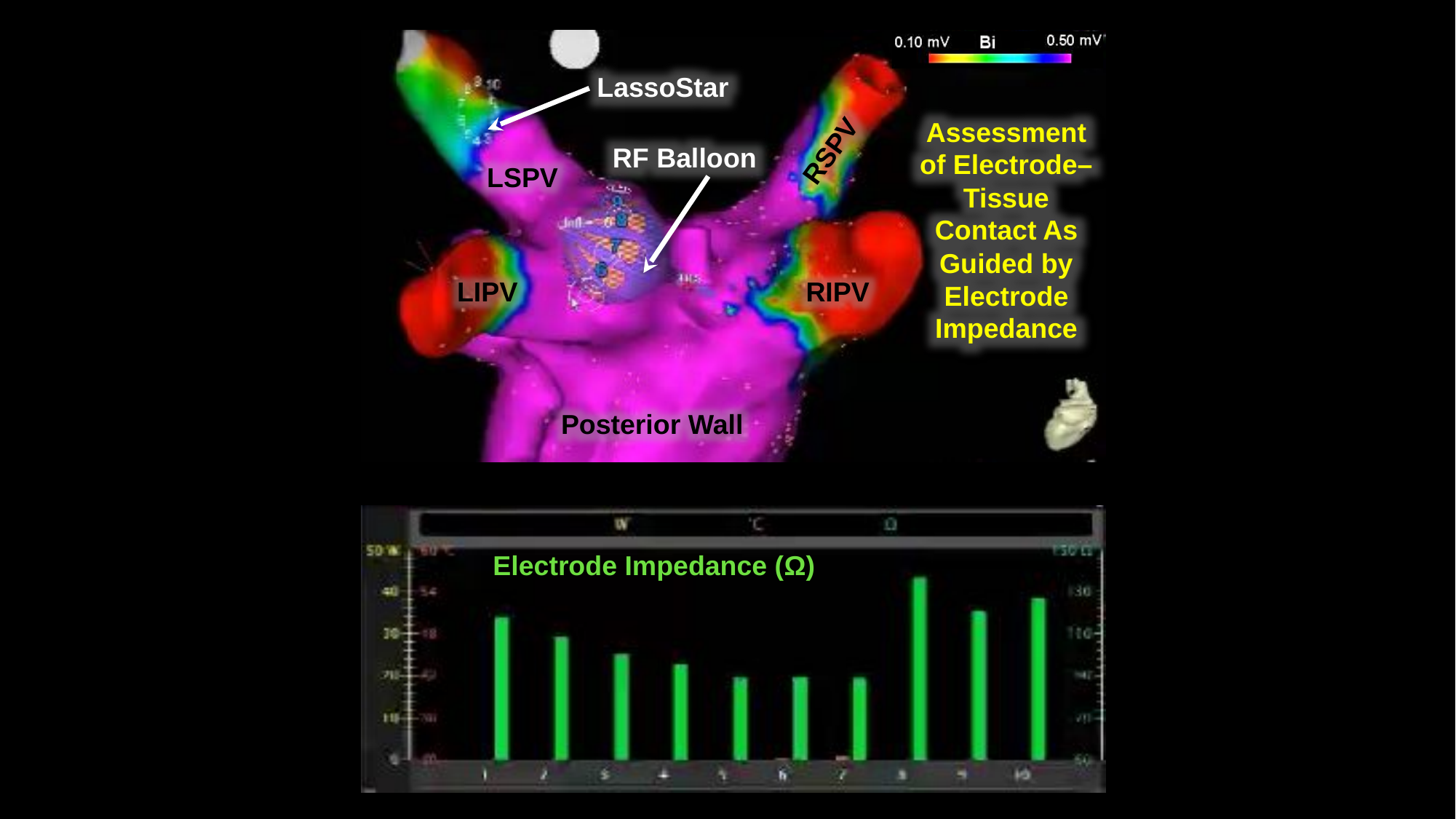

LassoStar
Assessment of Electrode–Tissue Contact As Guided by Electrode Impedance
RSPV
RF Balloon
LSPV
LIPV
RIPV
Posterior Wall
Electrode Impedance (Ω)
